# Optimization of Sirius Red-Based Microplate Assay to Investigate Collagen Production In Vitro

**DOI:** 10.3390/ijms242417435

**Published:** 2023-12-13

**Authors:** Csenge Szász, Domonkos Pap, Beáta Szebeni, Péter Bokrossy, László Őrfi, Attila J. Szabó, Ádám Vannay, Apor Veres-Székely

**Affiliations:** 1 Pediatric Center, MTA Center of Excellence, Semmelweis University, 1083 Budapest, Hungary; 2 HUN-REN-SU Pediatrics and Nephrology Research Group, 1052 Budapest, Hungary; 3 Department of Pharmaceutical Chemistry, Semmelweis University, 1092 Budapest, Hungary; 4 Vichem Chemie Research Ltd., 1022 Budapest, Hungary

**Keywords:** fibroblast, collagen, extracellular matrix, Sirius Red, functional, microplate, assay

## Abstract

Tissue fibrosis is characterized by chronic fibroblast activation and consequently excessive accumulation of collagen-rich extracellular matrix. In vitro microplate-based assays are essential to investigate the underlying mechanism and the effect of antifibrotic drugs. In this study, in the absence of a gold-standard method, we optimized a simple, cost-effective, Sirius Red-based colorimetric measurement to determine the collagen production of fibroblasts grown on 96-well tissue culture plates. Based on our findings, the use of a serum-free medium is recommended to avoid aspecific signals, while ascorbate supplementation increases the collagen production of fibroblasts. The cell-associated collagens can be quantified by Sirius Red staining in acidic conditions followed by alkaline elution. Immature collagens can be precipitated from the culture medium by acidic Sirius Red solution, and after subsequent centrifugation and washing steps, their amount can be also measured. Increased attention has been paid to optimizing the assay procedure, including incubation time, temperature, and solution concentrations. The resulting assay shows high linearity and sensitivity and could serve as a useful tool in fibrosis-related basic research as well as in preclinical drug screening.

## 1. Introduction

Fibrosis is an uncontrolled, pathological process, where constant profibrotic signals promote excessive fibroblast proliferation and overproduction of extracellular matrix (ECM), mainly collagen, which displaces the functional parenchyma and eventually leads to organ dysfunction. It can affect almost every organ and plays an important role in various disorders, such as chronic autoimmune and inflammatory diseases, graft loss after transplantation, or atherosclerotic plaque formation, making fibrosis a contributor to 45% of deaths worldwide [1,2]. Currently, the only approved antifibrotic therapeutic opportunities are the multikinase inhibitors nintedanib or pirfenidone, both targeting the activation and collagen production of fibroblasts. These drugs are used to treat idiopathic pulmonary fibrosis and have been shown to reduce disease progression rate, but they cannot prevent, stop, or reverse fibrosis; moreover, they have significant, mainly gastrointestinal, side effects that lead to therapy discontinuation [3]. Although a recent study has shown that the mean survival was significantly higher among patients receiving antifibrotic treatment, it still remained for approximately 5 years [4]. This global burden makes it imperative to reveal the exact pathomechanism of fibrosis, identify potential therapeutic targets, and develop effective antifibrotic compounds.

Fibroblasts are the main executor cells in fibrotic processes. Upon activation by cytokines, chemokines, and growth factors, they migrate to the site of tissue injury, start to proliferate, and produce ECM to form the mass of scar tissue, whose primary role is to ensure tissue integrity [5]. High-throughput microplate-based in vitro systems are essential to explore the exact pathomechanism and test the effect of antifibrotic compounds on different fibroblast functions. Cell viability or proliferation assays are used to monitor the increase in cell number through their enzyme activity, ATP production, or DNA replication [6]. Methyl thiazole tetrazolium (MTT) assay, a widely used method to investigate cell proliferation, belongs to the colorimetric assays and is based on the formation of the purple end-product formazan by mitochondrial succinate dehydrogenase enzyme in viable cells [7]. The reduction of apoptosis rate, also a sign of fibroblast activation, can be examined with the MTT dye-based lactate dehydrogenase (LDH) assay, referring to intracellular enzymes released from dead cells to the supernatant [8]. Recently, to substitute the scratch assay to evaluate the migration capacity of fibroblasts, our research group has developed a transient agarose spot (TAS) assay. This method is based on using agarose droplets as a temporary physical barrier to create a cell-free zone on the culture plate. The kinetics of gap closure and thereby the migration capacity of the examined cells can be determined by subsequent graphical analysis [9].

Although there are a variety of methods available to investigate collagen production in cell cultures, a simple, cost-effective, high-throughput assay is still lacking. Molecular biology techniques, such as immunocytochemistry, enzyme-linked immunosorbent assay, Western blot, polymerase chain reaction, or hydroxyproline assay, are expensive and often time-consuming methods that do not allow the simultaneous investigation of large numbers of samples. Of the traditional histological staining methods of collagen-rich ECM, Sirius Red has been proven to be the most specific and sensitive, compared to Masson’s trichrome or Van Gieson’s [10,11].

**Figure 1:**
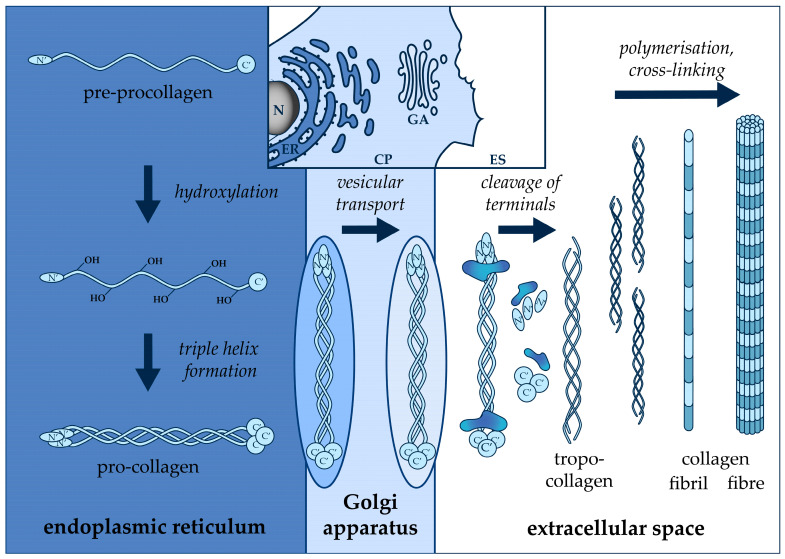
Collagen biosynthesis and maturation process. The **top middle** section of the figure shows the cell organelles with distinct shades of blue, involved in the post-translation modifications of collagen. Below, the schematic figure illustrates the intra- and extracellular steps of collagen fiber formation. Based on Veres-Székely et al. [16]. *N*: nucleus, *ER*: endoplasmic reticulum; *CP*: cytoplasm; *GA*: Golgi apparatus; *ES*: extracellular space.

In addition to the above-mentioned difficulties in labeling and quantifying total collagen, another factor complicates the development of a biologically relevant functional cellular assay. Collagen biosynthesis is a very complex procedure, consisting of multiple intra- and extracellular steps (Figure 1). After their transcription and translation, proline and glycine-rich pre-procollagen α-chains enter the endoplasmic reticulum, where post-translation modifications, including signal peptide cleavage, prolyl, and lysyl hydroxylation and glycosylation, result in the formation of triple helix pro-collagens. These water-soluble molecules leave the intracellular space by exocytosis through the Golgi apparatus, and then the N′ and C′ terminal propeptides are enzymatically removed. This modification allows the supramolecular organization of water-insoluble tropocollagen molecules into collagen fibrils by a quarter-staggered assembly, mediated by cellular integrins and fibronectin [12,13,14]. Further maturation and intra- and intermolecular crosslink formation result in a network of collagen fibers that are attached to the cell surface and surrounding tissue elements to support their organization [14]. These conditions are difficult to reproduce in vitro in order to ensure the uninterrupted maturation of collagens, and it must be considered when developing an appropriate functional assay based on a two-dimensional cell culture [1,15].

Currently, there are two Sirius Red-based commercial kits for collagen detection. Despite their relatively high prices, Sircol Collagen Assay (Biocolor, Carrickfergus, UK) and Sirius Red Total Collagen Detection Assay Kit (Chondrex, Woodinville, WA, USA) have several limitations. The original Sircol Collagen Assay was designed for the quantification of insoluble forms of collagens after collagen extraction by acid-pepsin digestion of tissue samples. This method, including the time-consuming sample preparation, takes a whole working day, and attention is drawn to certain interfering factors, such as albumin. Indeed, several studies warn of the shortcomings of the Sircol assay, recommending additional digestion and separation steps to improve its accuracy and usability [17,18]. A new version of Sircol was released at the time of the present study, dedicated to measuring soluble collagens. Although the manufacturer promises improved specificity, no reference is yet available. However, determination of total collagen production in vitro still requires a combination of different kits and overnight sample preparation and digestion. Chondrex provides a Sirius Red Total Collagen Detection Assay Kit for detecting collagen content in tissue and cell homogenate or cell culture medium. This method is very similar to Sircol, still requiring solubilization of solid samples by acidic or enzymatic digestion and also a concentration of a high amount of culture medium (1 mL)—in which the presence of serum causes high background values.

The aim of the present study was to develop a simple, fast, cost-effective in vitro microplate-based method to quantify the amount of mature collagens attached to the cell surface and that of immature, water-soluble or floating collagens secreted by the fibroblast into the culture medium, using the collagen-specific Sirius Red dye. In addition to developing staining procedures, we also optimized the setup of experiments, including potential adjuvants during the cell treatment. The resulting method is suitable for screening the efficacy of antifibrotic drugs on the collagen production of fibroblasts.

## 2. Results

### 2.1. Detection of Cell-Associated Collagens by Sirius Red Staining

**Figure 2:**
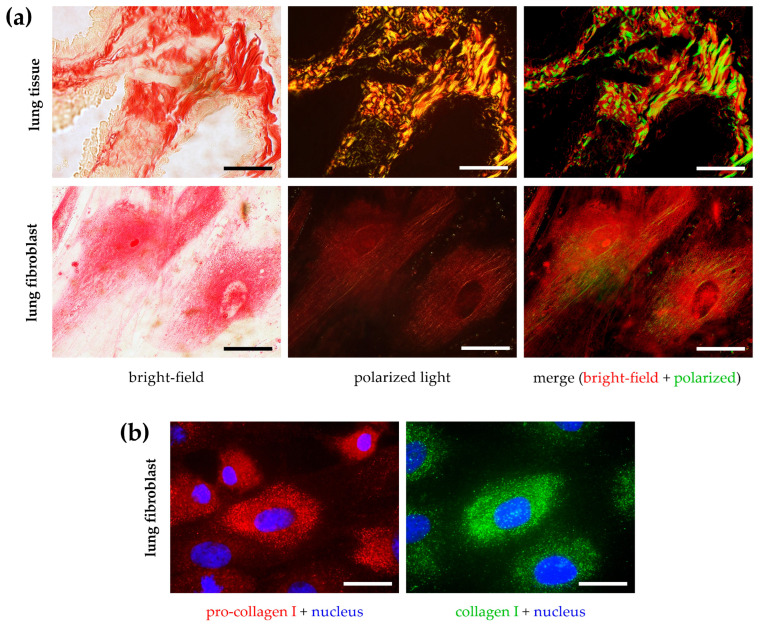
Detection of collagens in tissue and cell samples. (**a**) To visualize Sirius Red-stained fibrotic mouse lung tissue sections and CCD-19Lu lung fibroblasts, images were taken by bright-field and polarized light illumination microscopy. (**b**) To visualize immunostained CCD-19Lu lung fibroblasts using antibodies raised against pro-collagen I and collagen I, images were taken by fluorescence microscopy. Scale bar: 50 μm.

To visualize collagens in fibrotic lung tissue and cell culture, sections were stained with Sirius Red following the standard histological procedure, and then microscopic images were taken using bright-field and polarized light illumination techniques (Figure 2a). Large amounts of collagen deposition were detected in lung tissue samples, shown as red areas in bright-field images. Polarized light microscopy showed that the majority of collagens formed a complex system of fibers. In the case of adherent cell culture, the cytoplasm of fibroblasts showed Sirius Red positivity of which only a small fraction showed light scattering. Thin fibrils were mostly attached to the cell edges. Similarly, we found predominantly perinuclear and intracellular immunopositivity in fibroblasts, labeled with antibodies against pro-collagen or total collagen I (Figure 2b).

**Figure 3:**
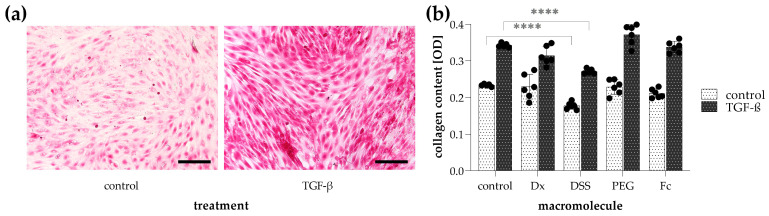
Detection of cell-associated collagens by Sirius Red staining on TGF-β-treated CCD-19Lu lung fibroblasts. (**a**) To visualize Sirius Red-stained cells, images were taken by bright-field microscopy. Scale bar: 200 μm. (**b**) The effect of macromolecular crowding on collagen production was investigated in the absence or presence of dextran (Dx), dextran sulfate sodium (DSS), polyethylene glycol (PEG), and ficoll (Fc). The amount of cell-associated collagens in Sirius Red-stained samples was determined by the optical density (OD) of their eluates. Results are presented as mean ± SD, dots represent individual values (*n* = 6). **** *p* < 0.0001 (two-way ANOVA with Dunnett’s test).

To investigate collagen production of fibroblasts, unstimulated and TGF-β-treated CCD-19Lu cells, grown in 96-well plates were stained with Sirius Red (Figure 3a). The amount of dye bound to cell-associated collagens was quantified by measuring the absorbance of their eluates. Treatment with TGF-β, however, resulted in increased Sirius Red positivity of fibroblasts, and the volume exclusion effect of macromolecular crowding, investigated by the addition of various polymers (dextran, dextran sulfate sodium, polyethylene glycol, or ficoll) to the cell medium had no impact on cellular collagen deposition (Figure 3b).

### 2.2. Detection of Collagens in Cell Culture Medium by Sirius Red Staining

**Figure 4:**
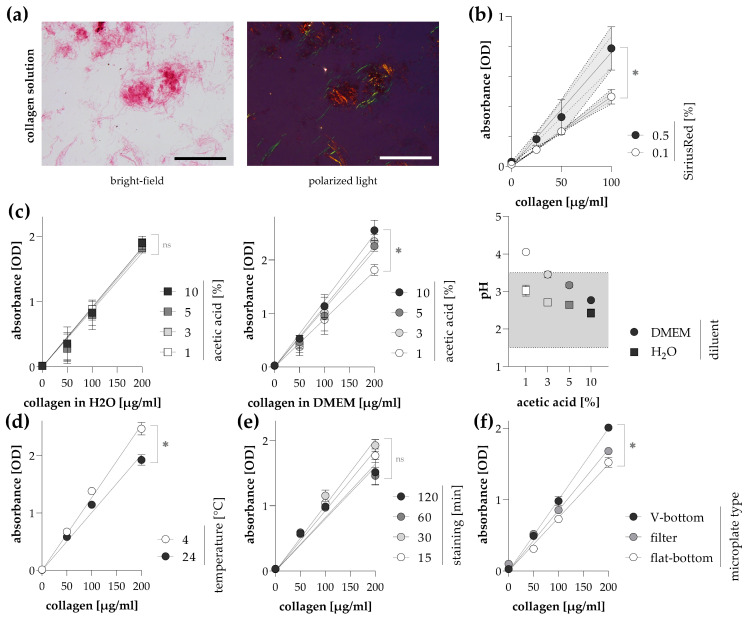
Detection of collagens in solution by Sirius Red staining. (**a**) To visualize Sirius Red stained collagen I solution (200 μg/mL diluted in culture medium), images were taken with a bright-field and polarized light illumination microscopy. Scale bar: 200 μm. The effect of (**b**) dye and (**c**) acetic acid concentration of the Sirius Red solution on its collagen labeling efficiency was investigated on collagen solution diluted with distilled water (H_2_O) or culture medium (DMEM). The pH of the resulting mixtures was also determined. The gray bar shows the optimal binding pH range of Sirius Red on tissue sections based on literary data. The effects of (**d**) temperature and (**e**) incubation time on the labeling efficiency were investigated on collagen solution diluted in a culture medium. (**f**) Different microplate types used during the washing steps were also compared. The labeling efficiency of Sirius Red-stained samples was determined by the optical density (OD) of their eluates. Results are presented as mean ± SD (*n* = 6). ns: non-significant, * *p* < 0.05 (linear regression).

To establish the staining method for the determination of soluble collagen amount in the cell media, collagen solutions were incubated with Sirius Red and the collagen-dye precipitates were separated by centrifugation. Microscopic observations revealed strong and specific labeling of fibrillar collagens (Figure 4a).

An increase in Sirius Red concentration in the staining solution resulted in higher absorbance of the eluates derived from the centrifuged collagen-dye pellets, but meanwhile, the intraassay variability was also multiplied (Figure 4b). The high deviation could be the consequence of auto-precipitation and the instability of dye crystals observed in the supersaturated solution. The impact of the acetic acid concentration of Sirius Red dye solution on the labeling efficiency was investigated in collagen solutions diluted with water or cell culture medium (Figure 4c). Low acid concentration resulted in decreased binding capacity of Sirius Red, presumably due to the buffer capacity of the medium, resulting in an alkalic pH shift of the solution mix from the optimal range.

We found that performing the staining and washing procedures at room temperature resulted in reliable and accurate data; however, the sensitivity of the Sirius Red assay can be further increased by cooling the reagents, thereby increasing the read signal (Figure 4d). Stable collagen-dye formation was detected at 15 min of staining of collagen solution, which could not be further increased by the elongation of incubation time (Figure 4e). When we compared the different microplate types used in the washing process, V-bottom plates gave the best results, whereas filters or flat-bottom plates with relatively large surfaces resulted in a loss of collagen-dye precipitates (Figure 4f).

**Figure 5:**
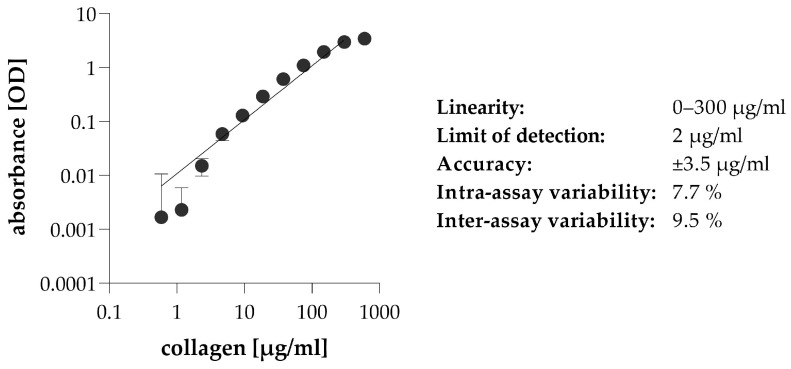
Validation of the established assay for collagen detection in solution by Sirius Red staining. To describe the efficacy of the method, collagen I solution series (0–600 ng/mL) diluted in cell culture medium (DMEM) were stained with 0.1% Sirius Red in 3% acetic acid, incubated at room temperature for 30 min, washed in V-bottom plate. The amount of collagens in Sirius Red-stained samples was determined by the optical density (OD) of their eluates. The efficiency of the method was described by determining linearity, sensitivity, and precision. Results are presented as mean ± SD (*n* = 6).

To validate the Sirius Red staining procedure of cell culture supernatants based on our results and impressions described above, a wide range of collagen dilution series were stained according to the chosen setup: 0.1% Sirius Red in 3% acetic acid, incubated at room temperature for 30 min, washed in V-bottom plate. The data obtained confirmed the good quality of our assay, characterized by a wide range of detection, high sensitivity, and very low (below 10%) intra- and inter-assay variability (Figure 5).

### 2.3. Optimization of In Vitro Experimental Setup to Detect Collagen Production of Fibroblasts

**Figure 6:**
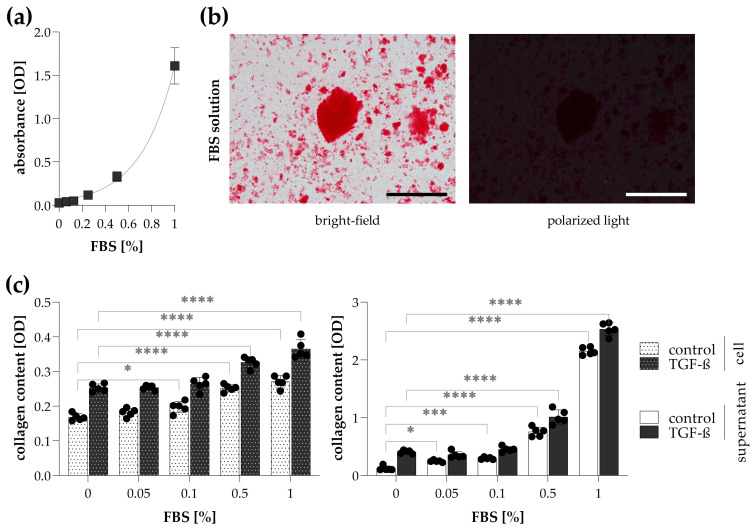
Effect of fetal bovine serum (FBS) on the detection of collagens in cell culture medium. (**a**) The non-specific binding of Sirius Red was investigated on FBS solution diluted in a culture medium. (**b**) To visualize Sirius Red precipitates in 1% FBS solution, images were taken by bright-field and polarized light illumination microscopy. Scale bar: 200 μm. (**c**) To investigate the effect of FBS on the detection of collagens, TGF-β-treated CCD-19Lu lung fibroblasts, and their culture supernatant were stained with Sirius Red. The amount of collagens in Sirius Red-stained samples was determined by the optical density (OD) of their eluates. Results are presented as mean ± SD, dots represent individual values (*n* = 5). * *p* < 0.05; *** *p* < 0.001; **** *p* < 0.0001 (two-way ANOVA with Dunnett’s test).

The effect of FBS concentration on collagen production of CCD-19Lu fibroblasts was investigated by simultaneous Sirius Red staining of the cells and their culture medium. We found that FBS itself formed flake-like precipitation with Sirius Red dye, resulting in a potential non-specific signal in cell culture medium samples (Figure 6a,b). Although the addition of FBS slightly increased the amount of cell-associated collagens, it also caused a high aspecific signal during the Sirius Red staining of cell culture medium samples (Figure 6c).

**Figure 7:**
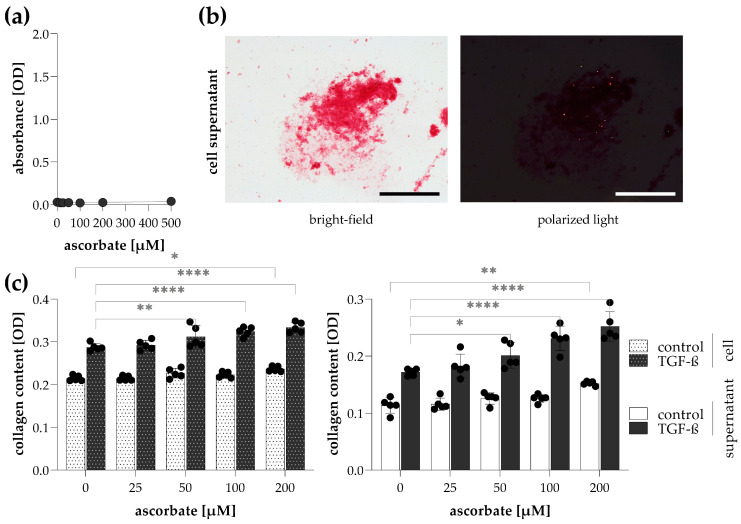
Effect of ascorbate on the detection of collagens in cell supernatants. (**a**) The non-specific binding of Sirius Red was investigated on an ascorbate solution diluted in a culture medium. (**b**) To visualize Sirius Red precipitates in a culture medium (200 μM ascorbate, TGF-β treatment), images were taken by bright-field and polarized light illumination microscopy. Scale bar: 200 μm. (**c**) To investigate the effect of ascorbate on the detection of collagens, TGF-β-treated CCD-19Lu lung fibroblasts and their supernatant were stained with Sirius Red. The amount of collagens in Sirius Red-stained samples was determined by the optical density (OD) of their eluates. Results are presented as mean ± SD, dots represent individual values (*n* = 5). * *p* < 0.05; ** *p* < 0.01; **** *p* < 0.0001 (two-way ANOVA with Dunnett’s test).

The impact of ascorbate on cellular collagen production was also investigated. Ascorbate solution formed no aggregates with Sirius Red dye (Figure 7a). Red precipitates separated from stained cell culture medium showed light scattering parts in polarized light (Figure 7b). The addition of ascorbate into the culture medium resulted in a dose-dependent increase in the collagen production of TGF-β-stimulated fibroblasts measured both on attached cells and in their culture medium (Figure 7c).

### 2.4. In Vitro Sirius Red Assay to Investigate the Efficacy of Antifibrotic Drugs

**Figure 8:**
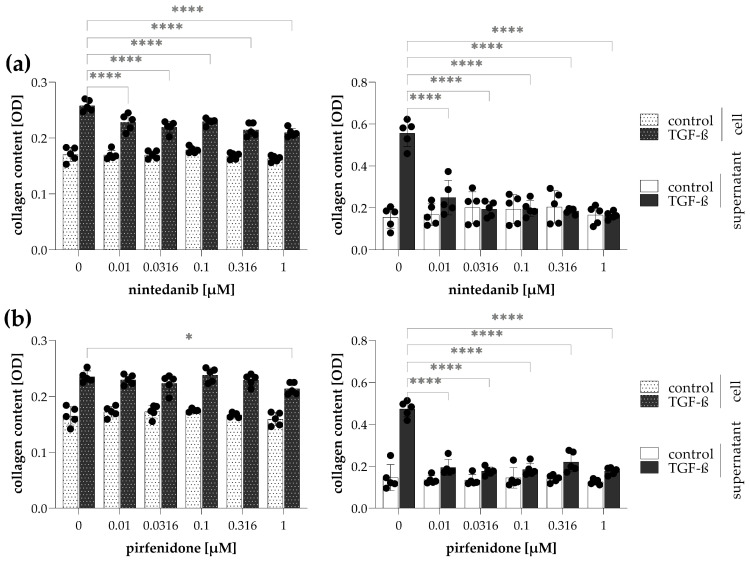
Sirius Red assay to screen the efficiency of antifibrotic drugs. To investigate the effect of (**a**) nintedanib or (**b**) pirfenidone, TGF-β-treated CCD-19Lu lung fibroblasts and their culture medium were stained with Sirius Red. The amount of collagens in Sirius Red-stained samples was determined by the optical density (OD) of their eluates. Results are presented as mean ± SD, dots represent individual values (*n* = 5). * *p* < 0.05; **** *p* < 0.0001 (two-way ANOVA with Dunnett’s test).

In representative experiments, we investigated the effects of nintedanib and pirfenidone on the collagen production of TGF-β-stimulated CCD-19Lu fibroblasts by simultaneous Sirius Red staining of the cells and their culture medium. We found that, although, treatment with nintedanib also reduced the amount of cell-associated collagens in a dose-dependent manner, with the effect of collagen levels being strong in culture supernatants, where it completely neutralized the inducing effect of TGF-β (Figure 8a). This difference was even more spectacular in the case of pirfenidone, which had little effect on cell-associated collagens, but even more so on those in cell culture medium (Figure 8b).

## 3. Discussion

To complete the assay arsenal that can be effectively used in fibrosis-related research, there is a need to develop a suitable method for the measurement of collagen production in a near high-throughput manner, in addition to fibroblast proliferation and migration assays [9]. In this study, we used lung fibroblasts to optimize the in vitro experimental setup to quantify collagens while considering the related challenges described above.

The collagen-detection method of our choice was based on Sirius Red dye. This traditional histological staining method has been used since the late 1960s as a potential substitution for Van Gieson’s picrofuchsin to detect connective tissue [19]. Initially, the labeling specificity of Sirius Red was investigated on amyloid plaques, but its usability proved to be inferior to its rivals [20,21]. In contrast, when Sirius Red was used in combination with picric acid, its specific collagen-binding affinity was found to be excellent; furthermore, the labeled collagen fibers showed intensely positive birefringence under polarized light [11]. There has been a debate about whether Sirius Red is able to differentiate between collagen types based on their color changes under polarized light, but this is more likely to be due to the orientation and thickness of the labeled fibers [10,22]. Indeed, it has been shown in several studies that Sirius Red stains various types (fibrillar I, II, III, V or network forming IV) of mature and pro-collagens with similar efficiency and also their native and digested forms [23,24,25]. Thanks to its above-mentioned properties, Sirius Red has become one of the most widely used histological staining techniques to detect fibrotic areas on certain tissue types [22,26,27,28,29]. As shown in Figure 2, Sirius Red staining can also be used to stain cellular collagens, not only on tissue sections.

The traditional analysis of Sirius Red-stained tissue samples is based on a scoring system and is evaluated by an experienced pathologist, where digitization and subsequent graphical analysis of sections is a well-established method that provides reliable and trustworthy results [30,31,32]. Data acquisition approaches for Sirius Red can be further extended to include its chemical properties. The dye molecule is characterized by an elongated structure with multiple anionic azo (sulfonic acid) groups, which allow Sirius Red to bind parallelly to the collagen triple helix, which contains mostly cationic amino acid residues [11]. By the way, this orientation of Sirius Red molecules arranged by the parallelly connected tropocollagens ensures the birefringence of fibrillar collagens, including type I and III [22,33]. As acidic conditions are required for the positive charge of collagen residues, the bound dye can be eluted with an alkaline solution, allowing its quantification based on optical density [18,25]. This approach of staining the fixed cells and then releasing the dye is much simpler and faster than the commercial kits discussed in the introduction, which require overnight sample preparation and solubilization.

In this study, we propose Sirius Red staining of fibroblast to determine the amount of cell-associated collagens. As shown in Figure 3a, fibroblast activation by treatment with profibrotic growth factor resulted in more intense staining and, consequently, higher absorbance of the eluted solution. However, as mentioned in the introduction, the maturation of collagens, which results in the cell-associated fiber form, is a complex multistep process, which is challenging to model in vitro. Indeed, due to the insufficient tropocollagen conversion, fiber arrangement, and cell linkage, a significant part of the biologically relevant collagen fractions cannot be measured as the cell culture medium is usually discarded during the staining procedure [1,15]. This is well illustrated by our microscopic images, which show that even after 7 days of culture, no significant collagen fiber alignment is found (Figure 2). Previously, it has been shown that the supplementation of cell culture medium with large macromolecules accelerated the mature collagen deposition in fibroblasts, which originally required several weeks under standard experimental conditions [34,35,36]. This phenomenon is known as the volume exclusion effect, based on the fact that in vivo both intra- and extracellular proteins are surrounded by other macromolecules, thereby reducing the actual, available volume of the aqueous environment [34,37]. Molecular crowding mediates protein interactions, including conformational changes, enzymatic reactions, and other biological processes. In spite of all these, we found no beneficial effect on the deposition of cell-associated collagens when large polymers (e.g., dextran, polyethylene glycol, ficoll) are added to the cell culture medium (Figure 3b), most probably due to the duration of the experiment. As time saving is also an important feature of the high-throughput in vitro assays, we looked for an alternative solution instead of extending the treatment from 48 h to 1–2 weeks.

Therefore, we preferred an alternative option to detect non-cell-associated collagens. As Sirius Red dye molecules bind to the collagen triple helix via ionic interactions, soluble pro-collagen, tropocollagen, and floating mature collagen forms can also be labeled (Figure 4a) [25]. The collagen-dye precipitates can be separated by centrifugation, and similarly to stained cells, pellets can be washed with an acid solution, thereby removing the excess dye, and then the bound dye can be eluted with an alkaline solution and its absorbance can be recorded. In the following, the optimization of collagen staining in a cell culture medium by Sirius Red will be discussed.

In the first step of our experiments, we investigated the effect of the staining environment on Sirius Red binding using a collagen solution. We found that the Sirius Red concentration we selected based on the original histological staining protocols [11] resulted in a stable, strong signal and that its enhancement was not expedient (Figure 4b). In contrast, a small modification of the acidity of the staining solution needed to be made, increasing the acetic acid concentration up to 3% (Figure 4c). This is most likely due to a pH shift of the sample-staining solution from the optimal range [11], caused by the buffer capacity of the cell culture medium. As previously discussed, an acidic environment is required for ionic interaction between Sirius Red and collagen molecules [25]. It has been previously shown that cooling staining reagents can improve the binding capacity of Sirius Red dye [38]. Similarly, we obtained higher absorbance values when collagen solutions were stained at 4 °C than at room temperature (Figure 4d). We found that providing low temperature during the implementation of the Sirius Red assay did not cause any difficulties in the treatment, and thus it was feasible to increase the sensitivity even for samples with low collagen concentration. We also examined the optimal incubation time of samples with Sirius Red solution. A stable signal was observed already at 15 min, which was not improved by longer staining (Figure 4e). On the basis of the literary data, long incubation times (usually 60 min) of fixed tissue samples are required due to the time-consuming diffusion of dye molecules into the tightly arranged collagen fibers, attached to the cells [11,24,39]. As the present assay is performed on 96-well culture plates, we compared different types of microplates to find the optimal one for multiple staining, centrifugation, and washing steps of cell culture supernatants. We found that the transparent V-bottomed plate was the most suitable for Sirius Red staining of solutions due to its small bottom surface, which results in well-formed precipitates, firmly attached to the bottom of the wells (Figure 4f). The efficacy and sensitivity of the final, optimized staining process is comparable to commercially available kits (Figure 5).

In the next step of the study, we optimized the in vitro experimental setup to investigate the collagen production of fibroblasts by Sirius Red staining on both cells and their culture medium, as described above. The majority of fibroblast-based methods to investigate collagen production is performed on prolonged or hyperconfluent cell culture, stimulated by TGF-β-treatment [35]. Therefore, in our experiments, cell seeding was followed by a proliferation phase triggered by high FBS concentration, which is one of the main components of the culture medium, containing large amounts of nutrients, ensuring cell growth [40]. However, it is known that FBS contains high amounts of albumin, a well-known carrier protein, rich in basic amino acid residues, which results in non-specific binding with various macromolecules, including Sirius Red [18,25,38]. Indeed, this was supported by our findings when staining culture media containing FBS with Sirius Red (Figure 6a,b). Meanwhile, our results showed that FBS supplementation during the fibroblast activation by TGF-β treatment had only a moderate effect on the amount of cell-associated collagens, but at the same time, it masked the specific signal detected in the culture medium (Figure 6b). Moreover, previously, it has been shown that collagen production can be increased by cultivating fibroblasts at high confluency and serum deprivation [41,42,43]. Therefore, we recommend the use of 0% FBS supplementation in cell treatments, in which case collagen production can be measured in a specific and sensitive manner in both cells and their medium by Sirius Red staining.

Finally, we investigated the effect of ascorbate supplementation on the collagen production of fibroblasts. Ascorbate, also known as ascorbic acid or vitamin C, is an essential cofactor of prolyl- and lysyl hydroxylase enzymes that perform the post-translational modification of collagen α chains, thereby ensuring their formation into triple helixes [44]. In addition, ascorbate has also been shown to affect collagen production and the transcriptional level by increasing the translation and the stability of collagen mRNAs [45,46,47]. The essential role of ascorbate on collagen biosynthesis is well illustrated by the typical symptoms of scurvy, a disease of ascorbate deficiency, affecting skin, gums, and joints among others due to collagen underproduction, leading to abnormal extracellular matrix formation in connective tissue [48,49]. We found that ascorbate increased the collagen production of fibroblasts in a dose-dependent manner, measured both on cells and in their culture medium (Figure 7). As ascorbate does not form precipitates with Sirius Red dye, we recommend 200 μM of ascorbate as a culture medium supplement during cell treatments.

As a result, we established an in vitro experimental system that allowed us to investigate the effects of various factors on collagen production. Our assay is based on a similar principle as commercially available Sirius Red-based kits; however, due to our specific recommendations for experimental setup, it finally provides more specific and sensitive results with a fraction of the cost (approx. 1/100) and time (approx. 2 h instead of a whole workday) required.

**Figure 9:**
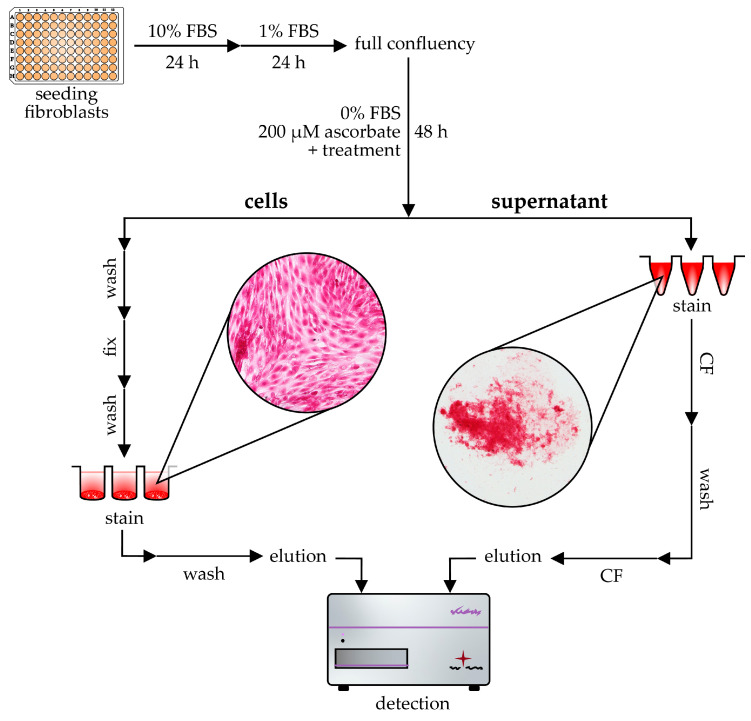
Schematic illustration of Sirius Red-based microplate assay to investigate collagen production in vitro. The figure shows the simultaneous staining and detection process of cell-attached and soluble collagens. *FBS*: fetal bovine serum; *h*: hours; *CF*: centrifugation.

Our representative measurements on lung fibroblasts showing the antifibrotic effect of the two FDA-approved drugs nintedanib and pirfenidone demonstrate the applicability of Sirius Red assay as an in vitro screening method (Figure 8). The combination of these two staining methods together provides biologically relevant and reliable data on cellular collagen production, simultaneously determining the amount of cell-attached and soluble form of collagens (Figure 9). The simplicity and cost-effectiveness of this microplate-based method further support its applicability in fibrosis-related research both in basic science and in preclinical drug development.

## 4. Materials and Methods

### 4.1. Cell Lines and Treatments

CCD-19Lu (#CCL-210, American Type Culture Collection, Manassas, VA, USA) human lung fibroblast cells were cultured in Dulbecco’s Modified Eagle Medium (DMEM) (Thermo Fisher Scientific, Waltham, MA, USA) supplemented with a 10% heat-inactivated fetal bovine serum (FBS) (Invitrogen, Waltham, MA, USA) and a 1% penicillin and streptomycin (Merck, Kenilworth, NJ, USA) mixture under standard cell culture conditions (37 °C, humidified, 5% CO_2_).

For in vitro experiments, CCD-19Lu cells were seeded into 96-well tissue culture plates (Sarstedt, Newton, MA, USA) at a density of 10^4^ cells/well (*n* = 5–6 wells/treatment group) and grown in DMEM with 10% FBS for 24 h, followed by 1% FBS in DMEM for an additional 24 h to achieve full confluence. Cells were then treated with 1 nM recombinant transforming growth factor beta 1 (TGF-β, #PHG9204, Thermo Fisher Scientific) diluted in FBS-free DMEM containing 200 μM ascorbate (Merck) for 48 h unless otherwise indicated.

To investigate the effect of volume exclusion, co-treatment with dextran 40 (Dx) (40 kDa, 50 mg/mL, Reanal Ltd., Budapest, Hungary), dextran sulfate sodium salt (DSS) (40 kDa, 100 μg/mL, MP Biomedicals, LLC, Irvine, CA, USA), polyethylene glycol (PEG) (20 kDa, 50 mg/mL, Merck) or ficoll 400 (Fc) (400 kDa, 50 mg/mL, Pharmacia Fine Chemicals, Uppsala, Sweden) were applied.

In representative experiments, co-treatment with 0.01–1 μM of nintedanib or pirfenidone (Vichem Chemie Research, Budapest, Hungary) was also investigated. Control cells were treated only with the respective solvents in equal volumes (TGF-β: 4 mM HCl, nintedanib, pirfenidone: DMSO).

### 4.2. Sirius Red Collagen Detection Assay for Cells

At the end of the in vitro experiments, after the removal of the culture medium, the cells were washed with 200 μL of phosphate-buffered saline (PBS) and fixed with 50 μL of Kahle’s solution, containing 26% ethanol, 3.7% formaldehyde, and 2% glacial acetic acid, at room temperature for 15 min. After another washing step with 200 μL of PBS, cells were stained by adding 50 μL of 0.1% Sirius Red (Direct Red 80) solution dissolved in 1% acetic. The plates were incubated at room temperature for 1 h, then wells were washed with 400 μL of 0.1 M HCl. Next, 100 μL of 0.1 M NaOH solution was added to each well to eluate the bound dye from the cell-associated collagens. Microscopic images were taken from Sirius Red-stained cells right before the elution step. All reagents were purchased from Merck. The absorbance of solutions was determined as optical density (OD) on 540 nm wavelength using a CLARIOstar microplate reader with MARS Data Analysis Software v4.01 (BMG Labtech, Ortenberg, Germany).

### 4.3. Sirius Red Collagen Detection Assay for Cell Culture Medium

A series of dilutions of rat tail-derived collagen type I (#A10483-01; Thermo Fisher Scientific) in distilled water or DMEM was used in the optimization steps of collagen staining in the culture medium. Centrifugation and washing steps were performed and compared in various 96-well microplates, including flat-bottom (Sarstedt), V-bottom (Thermo Fisher Scientific), or 0.45 μm low-binding hydrophilic polytetrafluoroethylene (PTFE) filter plate (Merck).

At the end of the in vitro experiments, cell culture medium with a volume of 100 μL was transferred into a V-bottom 96-well microplate (Sarstedt) and was stained by adding 50 μL of 0.1% Sirius Red solution dissolved in 3% acetic acid. The samples were mixed thoroughly and incubated at room temperature for 30 min. To separate the collagen-dye precipitates, the plates were centrifuged at 3000× *g* for 6 min. The unbound dye solution was aspirated with a multichannel pipette; thereafter, the wells were washed with 150 μL of 0.1 M HCl solution and subsequent centrifugation. To each well, 100 μL of 0.1 M NaOH solution was added to eluate the bound dye, thereafter samples were transferred into a transparent flat-bottom 96-well plate and their absorbance was measured as previously described. Microscopic images were taken from Sirius Red-stained collagen solutions or cell culture medium right before the elution step when collagen-dye precipitates were resuspended in an HCl solution and dripped onto a slide and covered by a glass coverslip.

### 4.4. Immunofluorescence Staining

CCD-19Lu cells were seeded into 4-well chambers (Sarstedt) at a density of 6 × 10^4^ cells/well and cultured in DMEM containing 10% FBS for 7 days. Subsequently, cells were rinsed with 500 μL of PBS, fixed and permeabilized with 300 μL of BD Cytofix/Cytoperm^TM^ (BD Biosciences, Franklin Lakes, NJ, USA) at room temperature for 15 min, then rinsed with 500 μL of BD Perm/Wash^TM^ (BD Biosciences). Cells were incubated with primary antibodies specific for either procollagen 1α2 (sc-166572; mouse, 1:100, Santa Cruz Biotechnology, Dallas, TX, USA) or collagen type I alpha 1 (sc-293182; mouse, 1:100, Santa Cruz Biotechnology) at room temperature for 1 h, washed with 500 μL BD Perm/Wash^TM^ for 2 min, incubated with goat anti-mouse Alexa Fluor^®^ 568-conjugated IgG secondary antibody (A11004; 1:500, Thermo Fischer Scientific) at room temperature for 1 h and washed again with 500 μL of BD Perm/Wash^TM^ for 2 min. Appropriate controls were performed omitting the primary antibodies to ensure their specificity and to avoid autofluorescence. Finally, slides were cover slipped using ProLong™ Gold Antifade Mountant (Thermo Fischer Scientific).

### 4.5. Microscopy

Microscopic images were taken with fluorescence, bright-field, or polarized light illumination techniques using the Olympus IX81 microscope system (Olympus Corporation, Tokyo, Japan) with 20× or 100× objectives.

Subsequent merging steps of corresponding image pairs were performed using ImageJ 1.48v software (National Institutes of Health, Bethesda, Rockville, MD, USA). Briefly, color images taken with bright-field and polarized light illumination from the same field of view were transformed into 8-bit format, then merged together using red (bright-field) and green (polarized) channels to visualize and compare the detected signals.

### 4.6. Statistical Analysis

Statistical evaluation of the data was performed using GraphPad Prism 9.1.2 software (GraphPad Software Inc., San Diego, CA, USA). Curves from the dilution series were compared by linear regression analysis. For the multiple comparisons of data from in vitro measurements, ordinary two-way ANOVA with Dunnett’s tests were used. Unless otherwise indicated, results are presented as mean ± SD of the corresponding groups. The tests applied, significance (*p*), and number of elements (*n*) are indicated in each figure legend.

Descriptive statistics were performed to describe the performance of ‘Sirius Red Collagen Detection Assay for Cell Culture Medium’. Linearity was determined based on the range of the collagen concentration for which a linear curve could be fitted, while regression (*R*^2^) < 0.95 was met. The limit of detection was determined based on the minimal collagen concentration at which the absorbance was significantly different from the baseline. Accuracy was defined as the mean difference between nominal and measured collagen concentrations in the linear range. Intra-assay variability was defined based on the average coefficient of variations from the samples of linear range measured in a single experiment. Inter-assay variability was determined as the coefficient of variation derived from the mean values of samples of linear range measured in six independent experiments.

### 4.7. Step-by-Step Protocol of In Vitro Sirius Red Assay

#### 4.7.1. Preparing Reagents

- Kahle’s fixative solution: 26% ethanol, 3.7% formaldehyde, and 2% glacial acetic acid diluted in distilled water
- Sirius Red solution for cell staining: 0.1% Direct Red 80 dissolved in 1% acetic acid containing distilled water
- Sirius Red solution for cell medium staining: 0.1% Direct Red 80 dissolved in 3% acetic acid containing distilled water
- HCl solution: 0.1 M HCl in distilled water
- NaOH solution: 0.1 M NaOH in distilled water

#### 4.7.2. Cell Culture and Treatment

- seed cells into 96-well plates to reach near-full confluence and culture them for 24 h in a culture medium containing 10% FBS
- change to medium containing 1% FBS for 24 h to reach full confluence
- change to medium containing 0% FBS, 200 μM ascorbate, and treat cells with the examined agents or factors for 48 h

#### 4.7.3. Sirius Red Staining of Cells

- transfer the cell culture medium into a V-bottom 96-well microplate
- carefully wash cells with 200 μL/well PBS
- fix cells with 50 μL/well Kahle’s solution at room temperature for 15 min
- wash cells with 200 μL/well PBS
- stain cells with 50 μL/well Sirius Red solution at room temperature for 1 h
- wash cells with 400 μL/well HCl solution
- elute collagen-bound dye with 100 μL/well NaOH solution

#### 4.7.4. Sirius Red Staining of Cell Culture Medium

- add 50 μL/well Sirius Red solution to the previously transferred 100 μL/well medium, mix thoroughly, and incubate at room temperature for 30 min
- centrifuge the plates at 3000× *g* for 6 min to precipitate the collagen-dye complex from the solution
- remove the remaining fluid carefully without touching the precipitates
- add 150 μL/well HCl solution, then centrifuge the plates at 3000× *g* for 6 min
- remove the remaining fluid carefully without touching the precipitate
- elute collagen-bound dye with 100 μL/well NaOH solution
- transfer the eluted solution into transparent flat-bottom 96-well plates for detection

#### 4.7.5. Detection

- measure the absorbance of the samples in a microplate reader at 540 nm wavelength
- either NaOH solution can be used as blank, or samples can be normalized on the background absorbance at 690 nm wavelength
- this method is applicable for the semi-quantitative comparison of collagen content between samples, but not for the exact quantification
- to quantify collagen content, collagen serial dilution in DMEM can be prepared and stained by the Cell Culture Medium protocol to create the standard curve

## Data Availability

Data are contained within the article.
